# Embossing Pressure Effect on Mechanical and Softness Properties of Industrial Base Tissue Papers with Finite Element Method Validation

**DOI:** 10.3390/ma15124324

**Published:** 2022-06-18

**Authors:** Joana Costa Vieira, António de O. Mendes, Marcelo Leite Ribeiro, André Costa Vieira, Ana Margarida Carta, Paulo Torrão Fiadeiro, Ana Paula Costa

**Affiliations:** 1 Fiber Materials and Environmental Technologies Research Unit (FibEnTech-UBI), University da Beira Interior, Rua Marquês d’Ávila e Bolama, 6201-001 Covilhã, Portugal; ant.mendes@ubi.pt (A.d.O.M.); malribei@usp.br (M.L.R.); fiadeiro@ubi.pt (P.T.F.); anacosta@ubi.pt (A.P.C.); 2 Aeronautical Engineering Department, São Carlos School of Engineering, University of São Paulo, São Carlos 05508-060, SP, Brazil; 3 Center for Mechanical and Aerospace Science and Technologies (C-MAST-UBI), University da Beira Interior, R. Marquês D’Ávila e Bolama, 6201-001 Covilhã, Portugal; andre.costa.vieira@ubi.pt; 4 Forest and Paper Research Institute (RAIZ), R. José Estevão, Eixo, 3800-783 Aveiro, Portugal; ana.carta@thenavigatorcompany.com

**Keywords:** embossing prototype, eucalyptus-based fibrous materials, FEM simulation, mechanical properties, pressure, softness, tissue paper

## Abstract

Embossing is a converting process in which the surface of a tissue paper sheet is changed under high pressure, allowing different functions. In this work, the authors intend to study how the embossing pressure affects the main properties of tissue paper, using a laboratory embossing system. An optimum pressure was achieved at 2.8 bar to this embossing laboratory set-up. The effect of pressure when densifying the paper sheet gives it a gain in mechanical strength but no differences in terms of liquid absorbency. The two embossing patterns present different behaviors but both evidence losses in mechanical and softness properties. On the other hand, the finite element method (FEM) does not show clear evidence of how the pressure affects the paper strength. For the deco die, it is possible to observe that the amount of yielding is slightly higher for lower pressure (2.4 bar), but this plasticity state parameter is very similar for 2.8 bar and 3.2 bar. For the micro die, FEM simulations of the manufacturing pressure do not show a considerable impact on the amount of plasticity state of the material; only for 3.2 bar, it shows a change in the pattern of the plasticity state of the paper during the embossing processes. In the end, to achieve a final product with excellent quality, it is important to make a compromise between the various properties.

## 1. Introduction

Tissue paper production in the last 10 years has increased mainly in Western Europe, having matched the production in North America, which until then was the leading producer of this kind of paper [1]. Nowadays, the importance of tissue paper should be highlighted.

Tissue paper can be defined as a cellulosic-based product (composed with virgin or recycled fibers) manufactured with low grammage, creped, and depending on the application, embossed (toilet paper, paper towels, and napkins). The most important properties of these papers are good flexibility, high surface smoothness, high bulk, and high capacity for liquid absorption. Tissue paper is creped, which causes a decrease in tensile strength in machine direction (MD) while increasing the sheet thickness, volume, and fiber-free ends. The creping process increases the elongation capacity in MD, resulting in a decrease in the Young modulus [2].

A roll of toilet paper is obtained at the end of the converting-line machine where one or more tissue paper mother reels are placed on cylindrical shafts. Different individual operations constitute the converting line, which includes the unwind, embossing, rewinder, perforation, LOG saws, and packaging [3].

Embossing is a forming process in which the surface of a substrate is changed under the influence of high embossing pressure. Embossing creates an image raised above the line of the paper surface and gives it a third dimension, the z-direction [4]. As a result, this process gives to the tissue sheet a certain texture. The embossing operation permits us different purposes. This process can be used to produce a flexibility effect, to exhibit an aesthetic pattern, or to promote bonding between several overlapping sheets of paper [5]. Embossing is the most prevalent process of all in the converting line, and it allows changing the paper’s intrinsic properties [6]. Embossers can perform this in a few ways. Embossing rollers can be steel–steel or rubber–steel. Steel rolls have engraved patterns, and when the embossed sheets are laminated (glued) they maintain a constant match [3,5].

Tissue paper experts have long recognized that the physical–mechanical properties of these products, such as grammage, thickness, strength, softness, and liquid absorption, are very important. Research and development has focused on improving each of these parameters without affecting the others simultaneously [7]. Balancing the different properties in paper converting is the main challenge to this industry. The embossing process contributes positively to bulk, liquid absorption, and softness (related to volume), and negatively to strength and surface softness. The lamination process, on the other hand, contributes positively to bulk (in some cases), absorption and smoothness (related to volume), and negatively to surface smoothness. The lotionizing process contributes to good softness at the cost of liquid absorption. Thus, for each process in the converting line, the impaired properties are available to be exchanged for others according to the requirements of the final product [6].

Grammage is one of the main properties of tissue paper. This is apparently a simple measurement but a very important one and gives us an initial indirect estimate of the amount of fiber present in the sample. Another equally important property is thickness, which indicates how bulky the base tissue paper is, or final product is. In the converting process, there is a potential loss of volume that can be compensated by the embossing process or by the development of a pattern that can compensate for this loss [8,9]. In fact, it is in the converting process that tissue paper thickness experiences the greatest changes, mainly caused by the embossing operation. So, the thickness of the final product is affected by the embossing pattern, embossing depth, nip pressure, lamination, and adhesiveness of the interlayer bonding. The embossing process is incorporated into the converting line to partially compensate for the loss of thickness in the converting process (web tensions used to control paper from unwinding to the rewinder and narrow nips along the machine) [9,10]. This is a critical point and the main reason why it is important to know and understand the thickness behavior during the entire conversion process. The embossing pattern, the handling of the final toilet paper, and the packaging strongly determine the final characteristics of this product, seen and felt by the final consumer [9,10].

Conventional tissue paper focuses on reducing the pressing step where the greatest loss of paper sheet volume occurs. Dry embossing is the most common method to increase bulk, but the volume created can be short-lived. A tight winding process produces a flatter sheet. More relevantly, the embossing pattern definition and volume are lost once the dry tissue paper is wetted again [2].

A paper with high strength or with a suitable elongation allows it to be processed at high speeds and/or create a high bulk after embossing. In opposition, it becomes more difficult to process the paper in the converting line (such as loss of sheet control, reduced speed, and/or lower embossing pressure) if its mechanical performance is not adequate [9,11].

As tissue paper has very low grammage, it therefore is more sensitive to variations in strength. In tissue paper, the main source of strength is the bonds between the cellulose fibers that compose it. Consequently, the strength of tissue paper is directly proportional to the number of fibers and their surface area. The type of cellulose fiber used is one of the most important factors when it comes to the strength of tissue paper. Generally, fiber blends are used to optimize the strength of the tissue paper sheet. Long fibers (softwood) contribute to the strength itself, while short fibers (hardwood) contribute to softness. Therefore, density has a direct influence on strength, as the greater the number of fibers in a given area, the greater the number of connections to be created [9,12].

Another critical factor regarding the tensile strength of tissue paper is its anisotropy. The strength of the sheet in MD gives us information about the tension that the sheet will be able to resist during the entire process without breaking. Specifically, in the embossing operation, the tensile strength and elongation in both directions determines the runnability of the sheet in the paper machine, where higher values allow greater prominence in the z-direction. All paper has a limit for embossing, where higher values generally result in greater volume, to the point where embossing will destroy the sheet strength properties and values fall below the minimum required to meet the machine’s production specifications, the conversion requirements, and/or the quality requirements of the finished product. As a result, in the embossing operation, it is essential to have the proper elongation and strength value to create the desired volume for a certain product. If the embossing takes place in the elastic zone of the paper sheet (minimum embossing), after embossing, it recovers and returns to its original form, losing the volume that the embossing process had just created. On the other hand, if embossing occurs near the breaking point (over embossing), the paper will be destroyed due to excessive pressure applied. In addition, the consequent loss in tensile strength may cause the failure of the paper sheet in the winding process [9,13].

Quantifying softness is a topic of many articles and controversies, both academically and industrially. However, all agree on the inverse and relatively linear relationship of the tensile strength of tissue paper with both mechanical and human panel softness [2,14]. Softness is also dependent on the embossing technology and patterns used to engrave the base tissue paper [15].

The quality of the final product, in addition to controlling its properties, also depends on the different physical operating parameters of the converting machine, such as temperature, humidity, and pressure. Embossing is a type of mechanical compression operation in the tissue paper manufacturing process, where pressure is a key parameter to produce a quality toilet paper [16]. From the previous work of Vieira et al. [17], the embossing operation was shown to have a negative impact on mechanical properties, being more pronounced for the micro embossing pattern. Additionally, this pattern also has a higher impact on the thickness and bulk increase. This article investigates the impact on the key properties of toilet paper of one of the main operational parameters in embossing: pressure. Using a laboratory embossing system, two different toilet base tissue papers were embossed at different pressures and after that tested for thickness, mechanical strength, and TSA smoothness. FEM analysis was also used to provide a better understanding of how the embossing patterns (deco and micro) affect the mechanical properties.

## 2. Materials and Methods

### 2.1. Materials

To perform this work, industrial toilet base tissue paper (only creped) from two different Portuguese factories was used. Both papers are composed of a mixture of bleached hardwood (*Eucalyptus globulus*) and softwood (*pinus*) kraft pulps, with hardwood being present in greater quantities. These two papers were selected because they have a very similar grammage and because the respective paper machines operate differently. The industrial base tissue paper designated by A was produced on a machine with a double headbox and steel creping blade, and the industrial base tissue paper designated by B was produced on a machine with a single headbox and ceramic creping blade. After the densification operation, these papers have the index d followed by the corresponding pressure value in their nomenclature, e.g., A_d 2.8_, and after the embossing operation it is the index me (micro embossing) and de (deco embossing), also followed by the corresponding pressure value, e.g., A_me 2.8_.

### 2.2. Methods

This work started by determining the grammage of the two industrial base tissue paper samples, which were measured using the paper tissue standard ISO 12625-6:2005 [18]. Then, the Fiber Tester from Lorentzen & Wettre was used to perform the morphological characterization analysis. Before executing the test, the samples were properly disintegrated, and the equipment calibrated according to the producer’s specifications. This test allows us to evaluate tissue paper samples in terms of length weighted in length of fibers (mm), width (μm), fines in length (%), and coarseness (mg/100 m).

**Figure 1:**
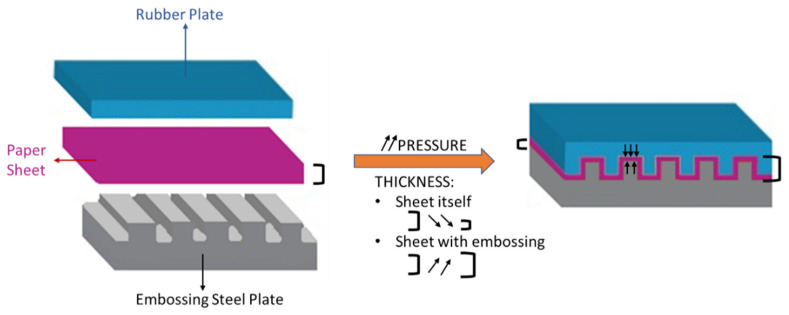
Scheme of the embossing process with pressure action effects.

Analyzing the embossing operation itself, the occurrence of two phenomena can be verified: the compression of the paper sheet and the engraving of the pattern. As can be seen in Figure 1, the thickness of the sheet itself decreases with increasing pressure, causing its densification. On the other hand, the overall thickness of the sheet due to embossing pattern engraving increases with increasing pressure.

Thus, the work continued to study these two effects. First, we proceeded to understand the densification effect of the sheet by compressing both base tissue paper samples with a flat steel plate and a rubber plate with a hardness of 60.3 ± 1.21 shore A (10 measurements along the rubber plate with a durometer PCE-DDA equipment), and the pressure was varied for values from 2.0 to 3.6 bar in 0.4 bar increments. In this procedure, the study of the liquid interaction with the different densified paper structures to evaluate spreading dynamics was also considered through an optical system [19], which has been used in previous studies [20,21,22,23]. The system works by ejecting droplets of approximately 0.5 μL of dyed water toward the surface of the densified paper samples and, simultaneously, a set of images of the droplets’ interaction was registered during a period of 3.0 s.

**Figure 2:**
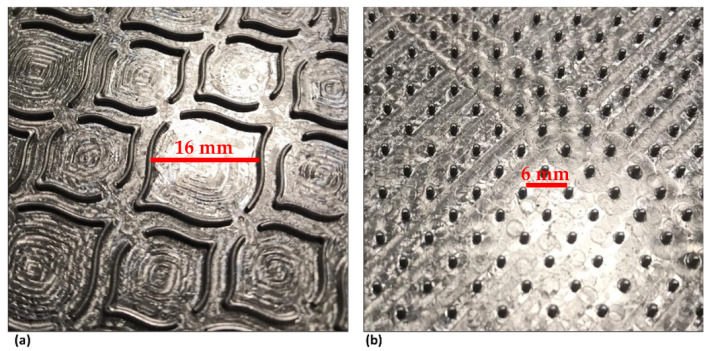
Photographs of steel embossing plates: (**a**) deco embossing, and (**b**) micro embossing.

Secondly, from the results obtained for densification, deco and micro embossing were carried out, also for both samples, using the same operating conditions but with steel plates with the embossing patterns, as shown in Figure 2. In this case, the pressure applied was 2.4, 2.8 and 3.2 bar. All sheets obtained by both methodologies were tested in terms of thickness/bulk, their mechanical tensile strength in the machine direction (MD), and in the transverse direction (CD). Tensile tests were conducted in a Thwing-Albert^®^ VantageNX Universal testing machine according to the tissue standard ISO 12625-4:2005 [24], and thickness and bulk were measured using a FRANK-TPI^®^ Micrometer according to the tissue standard ISO 12625-3:2014 [25]. In addition to these tests the apparent porosity (theoretical porosity) was determined by calculation using Equation (1):

P (%) = 100 × [1 − (ρ_sample_/ρ_cellulose_)] (1)

where ρ_cellulose_ is the cellulose density (assumed 1.6 g/cm^3^) and ρ_sample_ is the sample apparent density (in g/cm^3^), which is the inverse of the bulk (cm^3^/g) and calculated by the ratio between the grammage (g/m^2^) and the thickness (μm) of the sheet [26].

Both samples that were produced with an embossing pattern and the two initial base tissue papers were subjected to softness tests by the Tissue Softness Analyzer (TSA) from EMTEC^®^. The TP II algorithm and the QA I algorithm were used for the computation of the handfeel (HF), respectively. All the tested samples were prepared to meet the machine requirements.

### 2.3. Finite Element Method (FEM)

An FEM was proposed to improve the understanding of pressure on the paper manufacturing process. Two different die patterns were used for simulations (deco and micro), and their effects on the paper stress field and plasticity were analyzed regarding three different manufacturing pressures (2.4, 2.8 and 3.2 bar). As ABAQUS^TM^ does not have anisotropic plasticity, to model the paper plasticity an orthotropic elastic–plastic material model [27] was implemented as a user material subroutine for explicit simulations (VUMAT), linked to the commercial finite element software ABAQUS^TM^, to model paper plasticity. This material model allows considering paper anisotropic behavior, since paper response is highly dependent of fiber orientation [27]. The model assumes the decomposition of the strain tensor into elastic strain tensor plus plastic strain tensor (Equation (2)), and the volume is conserved.

(2) $$\varepsilon_{ij}=\varepsilon_{ij}^{e}+\varepsilon_{ij}^{p}$$

where $\varepsilon_{ij}$ is the total strain, $\varepsilon_{ij}^{e}$ is the elastic strain, and $\varepsilon_{ij}^{p}$ is the plastic strain.

The model uses the concept of an isotropic plasticity equivalent [28], which is a fictitious material that relates the orthotropic stress state to the isotropic stress state. The relation between the actual Cauchy stress tensor and the isotropic plasticity equivalent (IPE) deviatoric tensor is given in Equation (3).

(3) $$s_{ij}=L_{ijkl}\sigma_{kl}$$

where $s_{ij}$ is the deviatoric IPE stress tensor, $\sigma_{kl}$ is the Cauchy stress, and $L_{ijkl}$ is the fourth-order transformation tensor shown in Equation (4) for plane stress:

(4) $$L=\left[\begin{array}{ccc} 2A & C-A-B & 0\\ C-A-B & 2B & 0\\ B-C-A & A-B-C & 0\\ 0 & 0 & 3D \end{array}\right]$$

where the parameters *A*, *B*, *C*, and *D* are obtained from the experiments using the following equations (Equations (5)–(12)) [27]:

(5) $$A=\sqrt{1-12x^{2}}$$

(6) B=3(y−x)

(7) C=3(y+x)

(8) D=K12n(n+1)3

(9) $$x=\sqrt{\frac{\alpha^{2}}{24\left(3\alpha^{2}+\beta^{2}-4\beta +4\right)}\left(\beta +1-\sqrt{6\beta -3\alpha^{2}-3}\right)}$$

(10) $$y=\frac{\alpha }{4x}-A$$

(11) α=K332n(n+1)−K222n(n+1)

(12) β=K332n(n+1)+K222n(n+1)

The parameters $K_{ii}$ and n (in Equations (8), (11) and (12)) relate to the fit curve of the experimental data applying the Ramberg–Osgood methodology. For the MD direction tensile test, the Equation (13) is used:

(13) $$\varepsilon_{11}=\frac{\sigma_{11}}{E_{11}}+{\left(\frac{\sigma_{11}}{E_{0}}\right)}^{n}$$

For the CD direction, the Equation (14) is used:

(14) $$\varepsilon_{kk}=\frac{\sigma_{kk}}{E_{kk}}+{\left(\frac{K_{kk}E_{kk}}{E_{0}}\right)}^{n},\;k=2,3$$

Note that for Equation (14) the repeated indices do not mean the usual summation rule used in the indicial notation. Finally, the parameter $K_{12}$ is obtained using Equation (15).

(15) $$\gamma_{12}=\frac{\sigma_{12}}{G_{12}}+{\left(\frac{K_{12}\sigma_{12}}{E_{0}}\right)}^{n}$$

The Hooke’s law for plane stress small-strain linear elastic orthotropic material is defined by Equation (16):

(16) $$\sigma =C\varepsilon^{e}$$

where σ is the Cauchy stress tensor, C is the plane stress linear elastic orthotropic constitutive law, and $\varepsilon^{e}$ is the small-strain elastic tensor.

As plane stress was adopted for these simulations, the effect of the paper densification comes from the experimental tensile test for each configuration of die model and paper. The implementation of this model is similar to the well-known $J_{2}$ flow theory for isotropic materials using the backward-Euler algorithm. As mentioned before, this material model was implemented as a user material subroutine for explicit simulations linked to the commercial finite element software ABAQUS^TM^ version 6.14 (Waltham, MA, USA).

**Figure 3:**
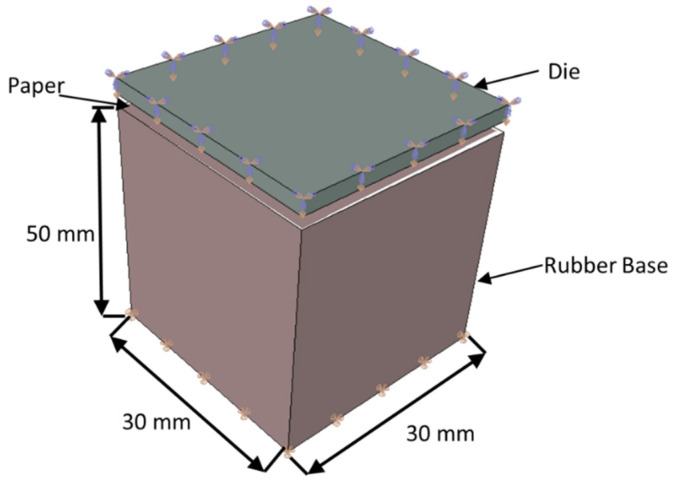
Model dimensions and boundary conditions.

The explicit solver was used to overcome convergence issues that are usual when using the implicit solver. On the other hand, the stable time increment is very small, resulting in long time simulations. The simulations were performed using a workstation with two intel Xeon E5-2630 8 cores (16 cores total with 32 threads) with 256 Gb RAM. The FEM dimensions and boundary conditions are presented in Figure 3. The die patterns’ dimensions are representative of the actual die dimensions to result in a reasonable computational cost and to keep the precision capability. The base dimension is the same, but it is thick enough to allow the deformation without severe interference in the die and paper kinematics. The paper follows the same dimensions of the die and basis.

All the displacement degrees of freedom are restricted in the bottom of the soft base. A prescribed displacement is applied on the top of the die. The other degrees of freedom are restricted. For the interactions between the parts, the model considers hard contact for normal behavior, and the tangential behavior is modeled with a penalty of 0.3 for the friction coefficient. The model has 473,694 elements for the simulation of micro die and 476,153 elements for the deco die. For both patterns, the paper was simulated using a 4-node reduced integration membrane element (M3D4R). The structured mesh has a total of 250,000 elements. For micro die, the 4-node reduced integration element (S4R) was used. The total number of elements is 7694 elements. The same element is used for the deco die, but for this die, 10,314 elements were used. Finally, the rubber base was modeled using 8-node tridimensional elements (C3D8). A total of 216,000 elements were used. This model uses 3 different types of material constitutive models: linear elastic for steel die (E = 200 GPa, μ = 0.33) and a hyperelastic isotropic material model for the rubber basis (C10 = 0.18 MPa and D1 = 0.0), and the paper was modeled using an orthotropic elastic–plastic user material model (E11 = 13.89 MPa, E22 = E33 = 4.23 MPa, μ = 0.33, and G12 = 2.1 MPa).

The tensile test in machine direction (MD) and cross direction (CD) were used to obtain the basic mechanical properties and the plastic parameters necessary for the IPE model (Equations (4)–(15)) used for these simulations. Then, the data points were used for the plastic parameter calculation, and a linear interpolation between those points were used to obtain the stress for a given plastic strain. It is important to mention that this procedure was applied for each combination of the paper densification and die model.

## 3. Results

**Figure 4:**
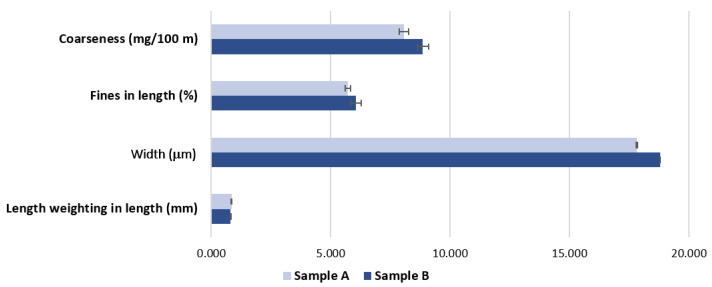
Morphological characterization of the two base tissue paper samples.

As intended, the grammage values obtained are very similar. For sample A, a grammage of 16.9 ± 0.01 g/m^2^ was obtained, and for sample B one of 16.7 ± 0.05 g/m^2^. Figure 4 presents the morphological characterization for the two samples, A and B.

To assess the impact of embossing pressure on tissue paper properties, we started by isolating the effect of pressure without the embossing patterns and how it impacted the properties and then, under the same conditions, what its impact is with the deco and micro embossing patterns.

### 3.1. Sheet Densification

**Figure 5:**
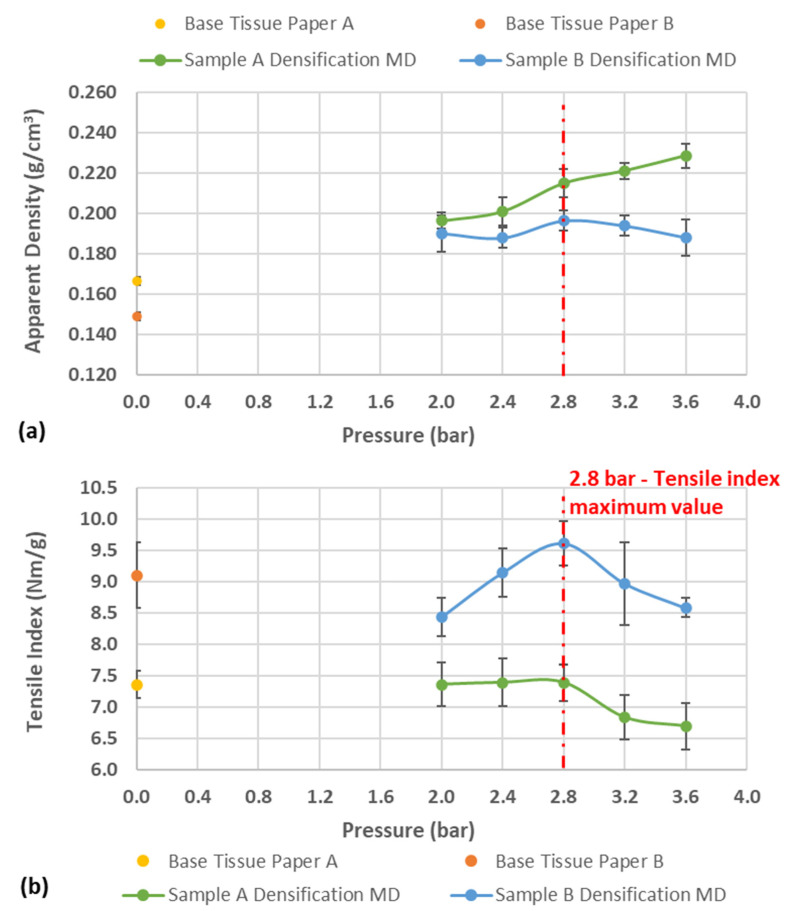
Results obtained for: (**a**) apparent density and (**b**) tensile index with the pressure increase in the base tissue paper densification of the samples A and B.

Figure 5 shows the behavior of apparent density and mechanical strength of the two samples for different pressures and without embossing patterns.

**Figure 6:**
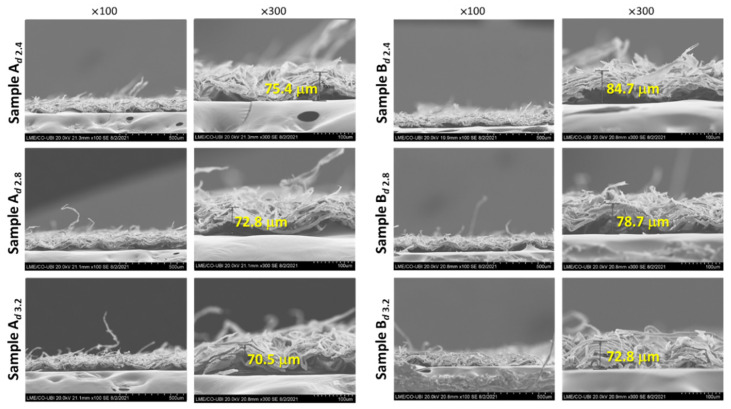
SEM images of samples A and B at two magnifications (×100 and ×300), at pressures of 2.4, 2.8, and 3.2 bar. The measurements presented in yellow corresponds to the height of the crepe wave.

Figure 6 allows the observation of the SEM images of samples A and B, at two magnifications (×100 and ×300) at peak pressures before and after it. The measurements in yellow correspond to the crepe wave height (μm) without any mechanical compression, which has a different interpretation than the thickness of tissue paper measured by the ISO standard.

**Figure 7:**
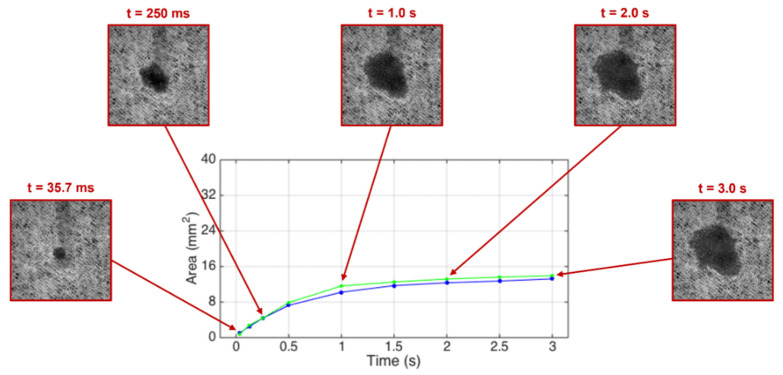
Water spreading area as a function of time for the non-compressed (blue line) and compressed (green line) base tissue paper sample A. The insets show the spreading area in different instants of time.

**Figure 8:**
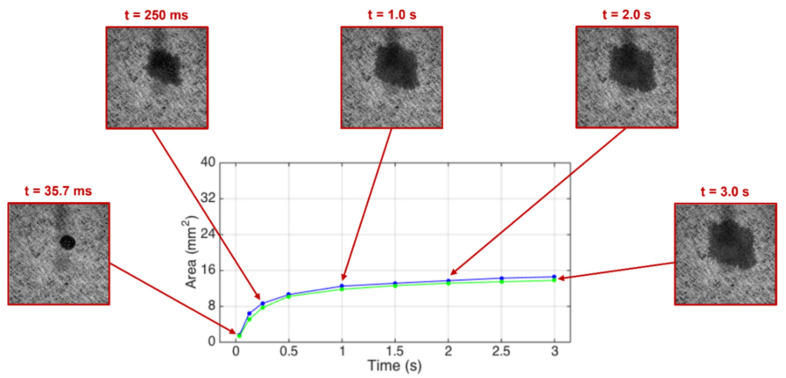
Water spreading area as a function of time for the non-compressed (blue line) and compressed (green line) base tissue paper sample B. The insets show the spreading area in different instants of time.

The water dynamic spreading area evolutions measured on the non-compressed base tissue papers and those compressed at the optimal pressure of 2.8 bar are presented in Figure 7 and Figure 8, respectively, for the samples A and B.

### 3.2. Sheet Embossing

The A and B sample results obtained for thickness, bulk, and apparent porosity for peak pressures, before and after, with deco and micro embossing, are presented in Table 1 and Table 2, respectively.

**Figure 9:**
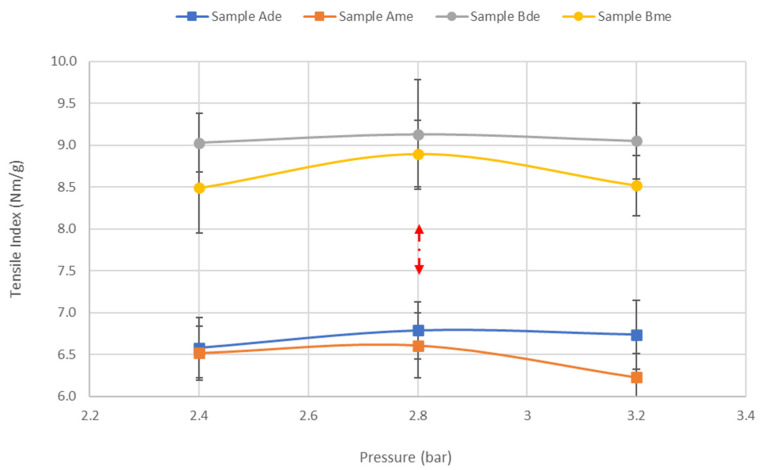
Results obtained for tensile index with the pressure increase in the samples A and B with deco and micro embossing.

The mechanical strength behavior with the pressure for the two embossing patterns in samples A and B are represented in Figure 9.

**Figure 10:**
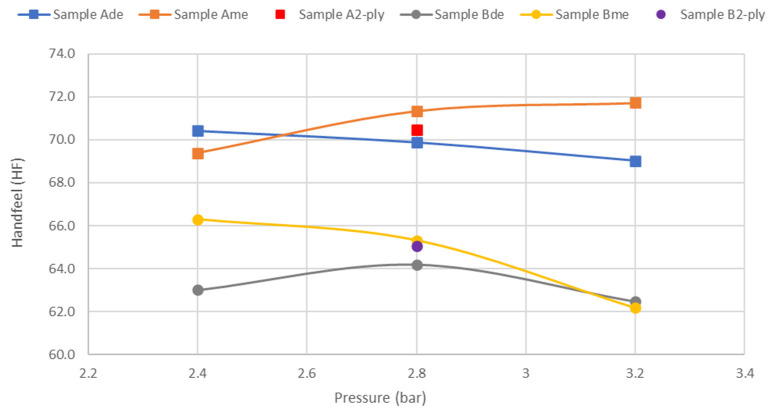
Results obtained for handfeel (HF) with the pressure increase in the samples A and B with deco and micro embossing.

Finally, Figure 10 shows the behavior of the important softness property with embossing pressure for the two patterns alone and combined in a final two-ply product.

### 3.3. Finite Element Method (FEM)

**Figure 11:**
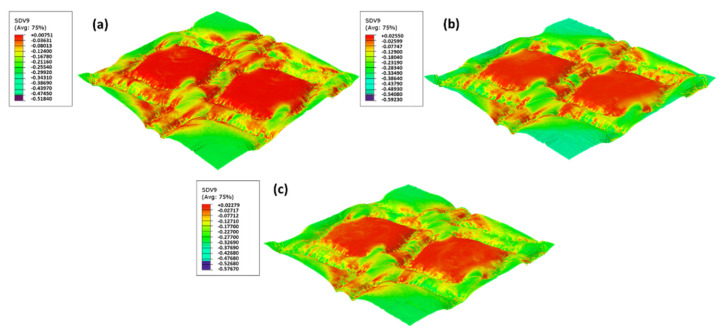
The yield function for deco die for different pressures: (**a**) 2.4 bar, (**b**) 2.8 bar, and (**c**) 3.2 bar.

The material model implemented as a VUMAT allows observing the plasticity state (SDV9) for each embossing pattern and manufacturing pressure and corroborates the experiments results for sample A. The influence of pressure in the manufacturing process impacts in the plasticity of the paper. For the deco die, the differences on yielding are presented in Figure 11.

**Figure 12:**
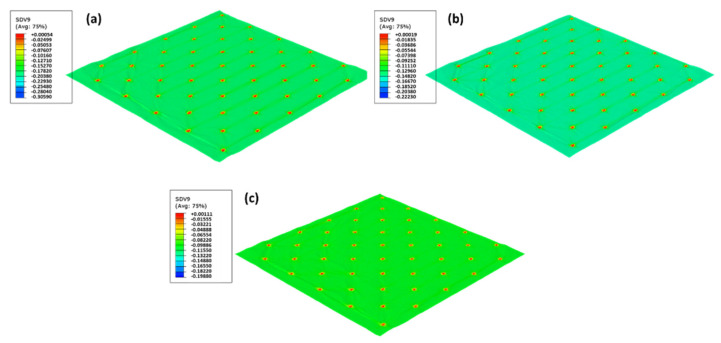
The yield function for micro die for different pressures: (**a**) 2.4 bar, (**b**) 2.8 bar, and (**c**) 3.2 bar.

For the microdie, the differences in yielding are presented in Figure 12.

## 4. Discussion

Analyzing Figure 4, for the length weighed in the length of the fibers, the values obtained are similar, but on the other hand, the coarseness and fiber width values are higher in sample B. These higher values indicate the existence of recycled fiber or broken process fiber in the fibrous composition of sample B. This information helps to evaluate the results of the remaining properties.

### 4.1. Sheet Densification

Examining Figure 5b, what immediately stands out is the peak at 2.8 bar pressure, where the maximum tensile index is obtained. Both samples show the same peak at the same pressure. On the other hand, looking at Figure 5a, there is an increase in apparent density when compared with the initial value, but above 2.8 bar the samples behave differently. This can be explained by the fact that sample B is not entirely composed of virgin fiber and therefore, from a pressure of 2.8 bar, the structure of the sheet itself is degraded by breaking the fiber bonds and opening the fibrous structure when the pressure is released, which means an increase in thickness.

Looking at the images in Figure 6, it is clear that in both cases, with increasing pressure, crepe waves decreased their height. In the case of sample B, the visible destruction of the crepe wave should be noted, with a redistribution of the fibers occupying the empty spaces of the crepe waves. These images corroborate the results discussed above, in which sample B has a more fragile fibrous sheet structure.

From this first part of the work, we can already see that there is an optimal pressure for the embossing process, where the effect of pressure when densifying the paper sheet can be advantageous, as the gain in mechanical strength can counterbalance the losses with the embossing operation.

The blue and green lines in the Figure 7 and Figure 8 show a very similar spreading dynamic evolution in time and within the same range, so there are no relevant differences between the non-compressed and compressed paper samples. However, it can be seen that the spreading evolution in the initial instants (t < 1.0 s) is faster for the base tissue paper B when compared with the base tissue paper A. It can be stated that this pressure (2.8 bar) does not affect the spreading of the liquids, but its structure densification is responsible for their different behaviors in the initial instants.

### 4.2. Sheet Embossing

Comparing Table 1 and Table 2, the structural properties of the paper sheet are more affected by the micro embossing pattern than by the deco, as expected. It should be noted that the structural property most affected is thickness, where for the micro embossing pattern an increase of 147% was obtained, while for the deco embossing pattern it remained practically constant with increasing pressure. Because the remaining structural properties are directly related to thickness, they did not undergo major changes for the deco embossing pattern, but for the micro embossing pattern the changes were in accordance with those obtained for thickness. These findings were verified in both samples, A and B.

It can be seen from Figure 9 that the peak at 2.8 bar pressure also occurs in all cases. Just as the micro embossing pattern is what highly affects the structural properties, it is also what impacts the most in the mechanical properties. Comparing the industrial toilet base tissue paper with and without embossing, for the micro embossing pattern, we obtained a loss of about 10% and 2% for sample A and B, respectively. Regarding the embossing deco pattern, as expected, this loss is smaller, obtaining about 8% and 0% for samples A and B, respectively. These results are in line with what was previously discussed, proving that the densification of the sheet was advantageous, so that the loss of mechanical properties by the embossing operation was minimized for the pressure of 2.8 bar.

Looking at Figure 10, the first statement that we can make is that the HF value for a two-ply final product with the two patterns will be between the HF values obtained for each pattern separately. Furthermore, the embossing deco pattern, in both cases, has lower HF values than the micropattern. Contrary to what happens with mechanical strength, it is not at 2.8 bar that the highest HF values are obtained. It is only for the case of the embossing deco pattern of sample B that the highest HF value is obtained for the pressure of 2.8 bar. Comparing the HF of industrial toilet base tissue paper with and without embossing, for the micro embossing pattern, we obtained a loss of about 9% and 12% for sample A and B, respectively. Regarding the embossing deco pattern, as expected, this loss is slightly higher, obtaining about 11% and 13% for samples A and B, respectively. It is necessary to make a compromise between the different properties in order to obtain the best possible result for the final product. Thus, there would be no effective gain in softness if the embossing process was operated at a pressure greater than 2.8 bar and the integrity of the fibrous structure of the paper sheet compromised.

### 4.3. Finite Element Method (FEM)

Despite the small differences for each pressure, it is possible to observe that the amount of yielding is slightly higher for the lower pressure (2.4 bar), but this plastic state parameter is very similar for 2.8 bar and 3.2 bar. Thus, the lower pressure model is more prone to fail since it has a higher area of greater yielding. For this simulation, the manufacturing pressure does not considerably influence the plasticity state. Only for 3.2 bar is a change in the pattern of the paper during the embossing processes shown. However, these differences are very small, and the finite element results do not allow to predict the differences in the strength of the paper.

## 5. Conclusions

This work allowed us to conclude that there is an optimal pressure for the embossing process, in which the mechanical strength is maximized without losing much of the softness value. With this laboratory embossing set-up, the optimum pressure achieved is 2.8 bar. The effect of pressure when densifying the paper sheet gives it a gain in mechanical strength but no differences in terms of liquid absorbency.

The authors can also conclude from this work that the two embossing patterns present different behaviors. The micro embossing pattern is the one that most affects the structure of the paper sheet, both in terms of structural properties and in terms of mechanical strength. On the contrary, the softness is more affected by the embossing deco pattern.

The work conducted with this laboratory embossing set-up came to corroborate the loss of mechanical properties and softness with the embossing process, regardless of the embossing pattern. Sample A was most affected in terms of mechanical properties, while sample B lost more in terms of softness, despite the use of any of the embossing patterns. Thus, the greater or lesser loss of mechanical or softness properties is dependent on the fibrous composition of each sample. In the end, it is essential to make a compromise between the diverse properties in order to achieve a final product with excellent quality.

Finally, the FEM allowed, for the deco embossing pattern, to understand the effect of pressure on the strength of the paper. On the other hand, for the micro embossing pattern, the FEM does not show clear evidence of how the manufacturing pressure affects the paper strength.

## Figures and Tables

**Table 1:** Thickness, bulk, and apparent porosity results for the base tissue paper samples A and B at different pressures with deco embossing.

	Sample A*_de_*				Sample B*_de_*			
Pressure (Bar)	Thickness (mm)		Bulk (cm^3^/g)	Apparent Porosity (%)	Thickness (mm)		Bulk (cm^3^/g)	Apparent Porosity (%)
	x	±σ			x	±σ		
0	0.102	0.001	6.01	89.2	0.112	0.002	6.71	90.3
2.4	0.100	0.001	5.89	89.0	0.117	0.002	7.00	90.7
2.8	0.101	0.002	5.99	89.2	0.111	0.001	6.62	90.2
3.2	0.102	0.001	6.01	89.2	0.110	0.002	6.60	90.2

**Table 2:** Thickness, bulk, and apparent porosity results for the base tissue paper samples A and B at different pressures with micro embossing.

	Sample A*_me_*				Sample B*_me_*			
Pressure (Bar)	Thickness (mm)		Bulk (cm^3^/g)	Apparent Porosity (%)	Thickness (mm)		Bulk (cm^3^/g)	Apparent Porosity (%)
	x	±σ			x	±σ		
0	0.102	0.001	6.01	89.2	0.112	0.002	6.71	90.3
2.4	0.152	0.013	8.98	92.8	0.209	0.019	12.49	94.8
2.8	0.203	0.014	12.01	94.6	0.258	0.021	15.44	95.8
3.2	0.252	0.010	14.88	95.6	0.270	0.018	16.14	96.0

## Data Availability

Not applicable.
